# Prevalence of Malaria among Adults in Ethiopia: A Systematic Review and Meta-Analysis

**DOI:** 10.1155/2021/8863002

**Published:** 2021-03-04

**Authors:** Fasil Adugna Kendie, Tamirat Hailegebriel W/kiros, Endalkachew Nibret Semegn, Melaku Wale Ferede

**Affiliations:** Department of Biology, Bahir Dar University, P.O. Box 79, Bahir Dar, Ethiopia

## Abstract

**Background:**

Malaria is one of the leading causes of mortality and morbidity in tropical and subtropical regions. The bulk of the global malaria burden is in sub-Saharan African countries, including Ethiopia. Malaria adversely affects the health of the peoples as well as the economic development of many developing countries including Ethiopia.

**Methods:**

This review article was reported according to PRISMA guidelines. Related published articles were searched from online public databases, such as PubMed, Google Scholar, and ScienceDirect. The search approach used to retrieve related articles were “prevalence,” “malaria,” “adults,” and “Ethiopia.” The quality of articles was assessed using Joana Brigg's Institute (JBI) critical appraisal checklist. The meta-analysis was computed using STATA version 14. The pooled prevalence estimates with 95% confidence interval were analyzed using a random-effect model, and the possible source of heterogeneity across studies was indicated through subgroup analysis, inverse of variance (*I*^2^), and time series analysis. The presence of publication bias was evaluated using funnel plots and Egger's regression test.

**Results:**

Out of 144 studies collected, only eight full-text articles were screened and included in the final quantitative meta-analysis. The pooled prevalence of malaria among adults in Ethiopia was 13.61%. Subgroup analysis based on types of malaria cases showed that the prevalence of malaria among symptomatic and asymptomatic adults was 15.34% and 11.99%, respectively. Similarly, regional subgroup analysis showed that the highest malaria prevalence was recorded in Southern Nations, Nationalities, and Peoples' Region (SNNPR) (16.17%) followed by Oromia Regional State (13.11%) and Amhara Regional State (12.41%). *Discussion and Conclusion*. The current systematic review and meta-analysis showed that the pooled prevalence of malaria among adults was found to be greater than the general population and nearly equal to pregnant women. Therefore, the current prevention and control measures, which are related to both vectors and parasites, should be strengthened.

## 1. Background

Malaria is a protozoan disease caused by *Plasmodium* parasites [1, 2], which is one of the leading causes of mortality and morbidity in many developing countries [3, 4]. An estimated 3.3 billion people are at risk of malaria worldwide [5], and it is a major health problem in tropical and subtropical regions [6]. World Health Organization estimates about 229 million new cases of malaria in 2019 in the world. Most of these malaria cases were in the WHO African region (94%). Likewise, there were 409,000 malaria deaths estimated in the globe. Most of these deaths occurred in the WHO Africa region (94%) [7]. Malaria is one of the major diseases of poor people in developing countries. The bulk of the global malaria burden is in sub-Saharan Africa, with the highest global cases and deaths. It is adversely affecting the health of the peoples as well as the economic development of many developing countries, particularly in sub-Saharan Africa [7–9].

Malaria causes serious complications, such as severe anaemia, cerebral malaria, acute renal failure, and hypoglycemia among infected individuals [10]. It is caused by five species of the genus *Plasmodium* [11]. Among these, four species including *P. falciparum*, *P. vivax*, *P. ovale*, and *P. malariae* are known to infect human beings in Ethiopia [12]. From the four *Plasmodium* species, *P. falciparum* is more severe in morbidity and mortality followed by *P. vivax* [13] with proportions of 60% and 40%, respectively [14].

Malaria is transmitted by the biting of an infected female *Anopheles* mosquito, which serves as a vector of *Plasmodium* species [1, 15]. There are more than forty species of *Anopheles* mosquitoes in Ethiopia [16–18] of which *Anopheles arabiensis, Anopheles funestus, Anopheles pharoensis*, and *Anopheles nili* are the malaria vectors. *A. arabiensis* is the primary malaria vector, while *A. funestus* and *A. pharoensis* are considered as secondary vectors in Ethiopia [19, 20].

In Ethiopia, malaria is one of the main public health and economic problems. The distribution varies from place to place depending on climate, rainfall patterns, and altitude [8, 21]. It is a major concern in the country, and it is one of the leading causes of morbidity and mortality. In Ethiopia, 75% of the landscape areas below 2000 m above the sea level are affected by malaria cases [22]. The major epidemics occur cyclically in every 5–8 years in Ethiopia, but focal epidemics are occurring every year [23]. Ethiopia has a population of above 100 million, of which approximately 68% of the population is at risk of the disease [22, 24]. About 2.9 million cases of malaria and 4,782,000 related deaths have been reported annually, and the rate of morbidity and mortality dramatically increases during epidemics [22, 25].

Ethiopia is one of the countries that have implemented the revised strategies to control malaria. Among these, indoor residual spraying (IRS) and long-lasting insecticidal nets (LLINs) are the most important in malaria prevention and control strategy [26]. Additionally, introduction of rapid diagnostic tests at community level and adaptation of artemisinin-based combination therapies (ACTs) are also practiced in Ethiopia [27]. Despite the massive efforts to implement these strategies, malaria continues to cause significant morbidities and mortalities in the endemic foci of Ethiopia. On the other hand, the developments of insecticide resistance on IRS and LLINS in different parts of the country have its own impact to control the main malaria vectors. In Ethiopia, the development of resistance in different insecticide groups on *A. arabiensis* was reported by Yewhalaw and his colleague [26], Abate and Hadis [28], Massebo and Lindtjørn [29], and others.

Malaria causes much damage to the health and socioeconomic development of the country [22]. The disease is more severe in children and pregnant women in the country [7]. Although several malaria prevalence studies in different parts of Ethiopia have focused on all age groups of individuals, only a few studies have indicated that adulthood malaria is still one of the major public health problems in the country. To date, however, the national estimate of adulthood malaria in the country is not known.

Systematic review and meta-analysis generate concrete evidence in which the evidence may help policymakers and program managers to design appropriate intervention to control and minimize the negative consequences of adulthood malaria. Furthermore, there is no published systematic review and meta-analysis that generated the pooled estimate of the prevalence of malaria at adulthood stage in Ethiopia. Therefore, the aim of this study was to determine the pooled prevalence of adulthood malaria in Ethiopia from studies conducted between 2010 and May 2020.

## 2. Methods

### 2.1. Search Strategy

This review article was reported according to the Preferred Reporting Items for Systematic Reviews and Meta-Analyses (PRISMA) guidelines. Related published articles were searched from online public databases such as PubMed, Google Scholar, and ScienceDirect, which reported the prevalence of malaria among adults in Ethiopia from 2010 to May 2020.

The search terms were used in agreement with the Medical Subject Heading (MeSH) using the arrangement of key words which were used to select related research articles. The search terms were used separately and in combination using Boolean operators like “OR” or “AND.” The search approach used to retrieve related articles was as follows: ((Prevalence) OR prevalence [MeSH Terms]) AND malaria) OR malaria [MeSH Terms]) (AND adults) OR adults [MeSH Terms]) AND Ethiopia) OR Ethiopia [MeSH Terms]). The repeated articles were excluded in the systematic and meta-analysis review. The software EndNote version X5 (Thomson Reuters, New York, NY) was used to arrange references and remove repeated references.

### 2.2. Eligibility Criteria

#### 2.2.1. Inclusion Criteria

Studies published in the peer-reviewed journals, which reports the prevalence of malaria among adults, were included. All studies were original research articles published in English and contained the basic information concerning sample size, diagnostic methods, prevalence, and status of malaria infection among adults in different parts of Ethiopia. Moreover, studies that have been carried out in health institution and community-based settings, symptomatic or asymptomatic individuals, and coinfections (malaria and HIV/AIDS) as far as they reported malaria among adults were included this review.

#### 2.2.2. Exclusion Criteria

Studies conducted among all age groups, pregnant women, children, unknown malarial detection methods, unknown sample size, and lack of clear figure about infected cases were excluded from this review.

### 2.3. Search Methods and Quality Assessment

Two authors (FA and TH) individually conducted a search in PubMed, Google Scholar, and ScienceDirect using the keywords. The searched articles were screened by the title and abstract to transfer the articles in the full-text review. The quality of articles was assessed using Joana Brigg's Institute (JBI) critical appraisal checklist for simple prevalence [30]. The two authors (FA and TH) independently assessed the quality of journals included in this review. The differences in the inclusion and quality of individual articles between the two authors were resolved by discussion with the third author (EN).

### 2.4. Data Extraction

Data extraction protocol was developed by two authors (FA and TA) and evaluated by EN and MW. This extraction protocol consists of name of the first author, year of publication, study area (region), study group, study design, sample size, prevalence of malaria, prevalence of *P. falciparum*, the prevalence of *P. vivax*, prevalence of mixed infection, and type of the diagnostic method used.

### 2.5. Data Analysis

Eligible primary studies were extracted, entered into Microsoft Excel, and then exported to STATA version 14. Forest plots were used to estimate the pooled effect size and effect of each study with their confidence interval (CI) and to provide a visual image of the data. The degree of heterogeneity between the included studies was evaluated by the inverse of variance (*I*^2^) [31]. *I*^2^ values of 25%, 50%, and 75% were assumed to represent low, medium, and high heterogeneity, respectively. Due to the observed high heterogeneity across studies, we used a random effect model to assess pooled estimate. The analysis between the subgroups was carried out regarding the type of cases. Small study effect and publication bias across studies were evaluated by the funnel plot symmetry subjectively and Egger's regression test objectively.

## 3. Results

### 3.1. Selection and Characterization of Included Studies

**Figure 1:**
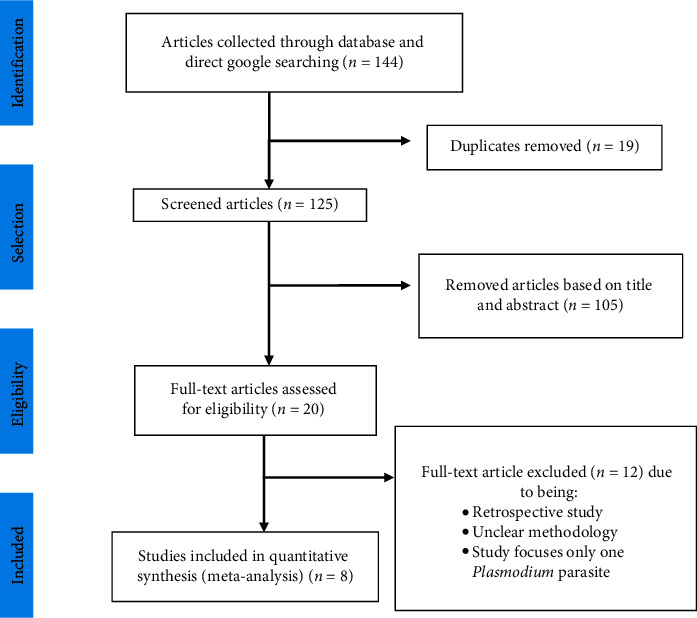
PRISMA flow diagram showing the selection process of eligible studies for this review, 2020.

This systematic review contains published articles on the prevalence of malaria among adults. A total of 144 articles were retrieved from online databases using manual searching. From these studies, 19 articles were excluded due to duplication records. From the remaining 125 articles, 105 of them were excluded by evaluation of their title and abstract. The remaining 20 articles were eligible for full-text assessment. From the 20 eligible articles, 12 studies were excluded with specific exclusion criteria such as being retrospective study, studies with unclear methodology, and studies focusing only one *Plasmodium* parasite. Finally, only eight full-text articles were screened for eligibility and included in the final quantitative meta-analysis (Figure 1).

Eight original full-text articles were included in this systematic review and meta-analysis. A total of 7895 study participants were tested for malaria infection from the eligible articles for this review. Among these, 775 were positive for malaria infection (377, 368, and 30 for *P. falciparum*, *P. vivax*, and mixed infections, respectively) (Supplementary file 2). Seven of the eight articles were used cross-sectional design, and the remaining one study used longitudinal research design. The sample sizes included in the eligible studies were ranged from 385 [32] to 3638 [33] populations. All studies were used for microscopic examination except for one study, which used RDT. All studies included in this review were carried out in three regions: 37.5% from Amhara Regional State, 37.5% from Oromia Regional State, and the remaining 25% from Southern Nations and Nationalities Peoples' Regional State (SNNPR). Unfortunately, there was no study found from other Regional States and city administrations such as Tigray, Afar, Somali, Gambella, Harari, Benshangul Gumuz, Addis Ababa, and Dire Dawa city administrations.

### 3.2. Quality Assessment

All studies were evaluated with nine criteria of the JBI quality assessment tool for the prevalence studies included in this review. The result of the analysis showed that the included studies had low risk of bias because its total score is 72%, which is greater than 50% (Supplementary file 1).

### 3.3. Prevalence of Malaria among Adult Stages

**Figure 2:**
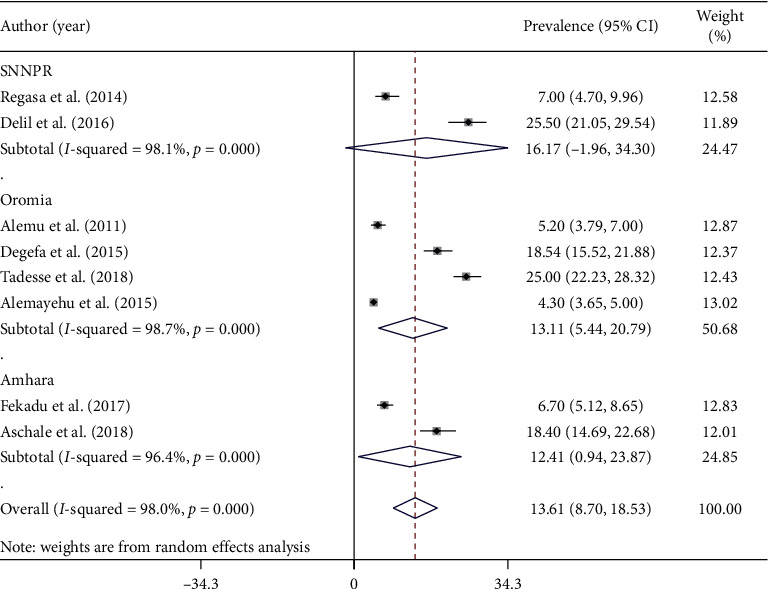
Forest plot showing the combined malaria pooled prevalence estimate among adults by regions of study in Ethiopia, 2020.

Eight articles were selected for this systematic review and meta-analysis, which were used to estimate the pooled prevalence of malaria among adults. The minimum prevalence of malaria was 4.3% observed in Oromia Regional State of different hospitals and health center [33], while the maximum malaria parasites was 25.5% observed in SNNPR of Hadiya Zone Health Centers [34]. The pooled prevalence of malaria among adults in Ethiopia was 13.61% (95% CI: 8.70–18.53) (Figure 2). High heterogeneity (*I*^2^ = 98% and *p* value ≤ 0.001) across studies was observed in the analysis.

### 3.4. Subgroup Analysis Based on Symptoms and Regions of Study

**Figure 3:**
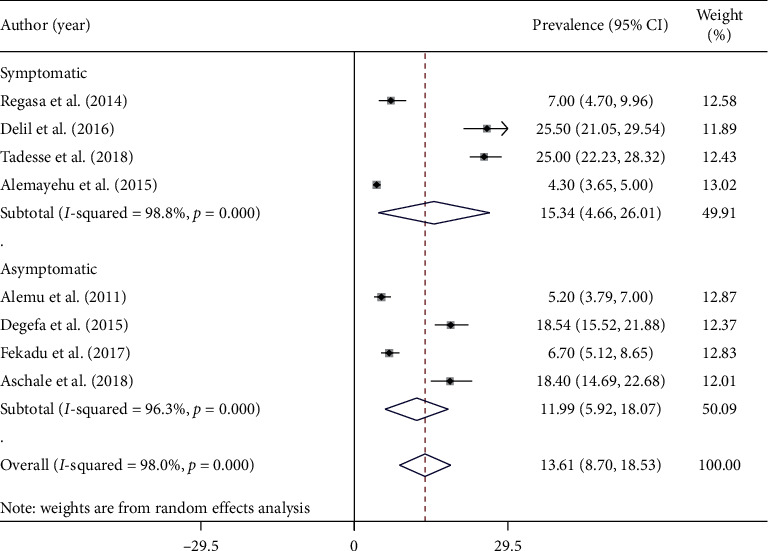
Forest plot showing the combined malaria pooled prevalence estimate among adults by types of study participants in Ethiopia, 2020.

Subgroup analysis based on types of malaria cases showed that the prevalence of malaria among symptomatic and asymptomatic adults was 15.34% (95% CI: 4.66–26.01) and 11.99% (95% CI: 5.92–18.07), respectively (Figure 3). Similarly, regional subgroup analysis showed that the highest malaria prevalence was recorded in SNNPR (16.17%) (95% CI: −1.96–34.30) followed by Oromia Regional State (13.11%) (95% CI: 5.44–20.79), and the least was documented in Amhara Regional State (12.41%) (95% CI: 0.94–23.87) (Figure 2).

### 3.5. Prevalence of Malaria Parasite Species among Adults

**Figure 4:**
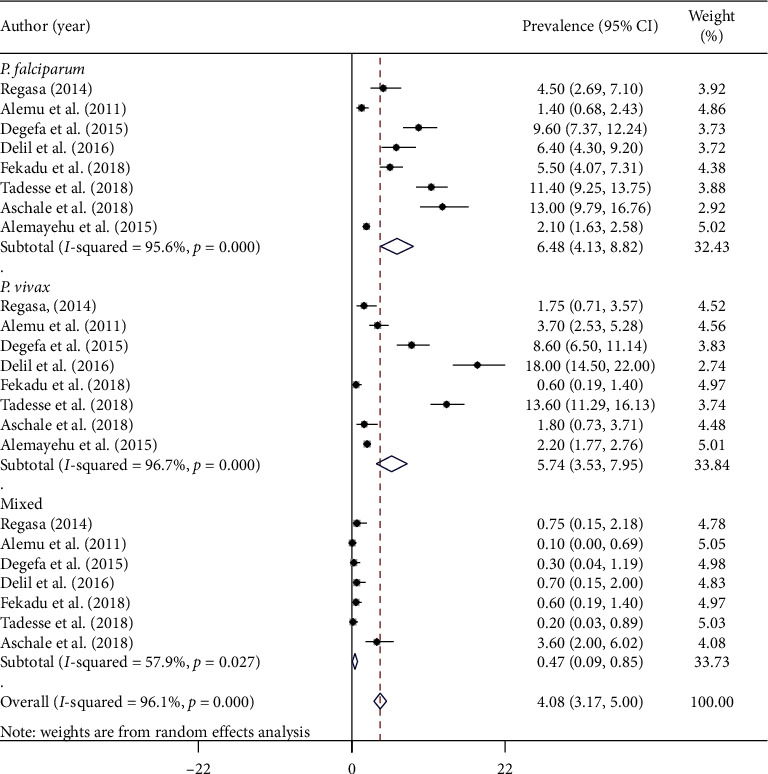
Forest plot showing malaria pooled prevalence estimate among adults by *Plasmodium* parasites in Ethiopia, 2020.

The prevalence of *Plasmodium* parasite species was compared between studies among Ethiopian adults. All of the studies [8] included in this review reported the prevalence of *P. vivax* and *P. falciparum* infection, while the prevalence of mixed infection was reported from seven studies. The overall pooled prevalence of *Plasmodium* species was as follows: *P. falciparum* (6.48%) (95% CI: 4.13–8.82), *P. vivax* (5.74%) (95% CI: 3.53–7.95), and mixed infection (0.47%) (95% CI: 0.09–0.85) (Figure 4).

### 3.6. Publication Bias across Studies

**Figure 5:**
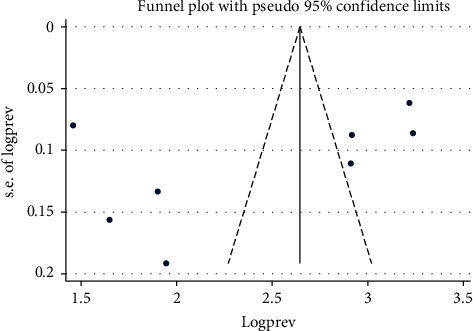
Funnel plots indicate absence of publication bias across studies included in this review in Ethiopia, 2020.

The presence of publication bias was evaluated subjectively using funnel plots symmetry and objectively using Egger's regression test. The symmetrical distribution of the funnel plot indicated the absence of publication bias across studies (Figure 5). The result of Egger's regression test (*pvalue* = 0.262) indicated there was no evidence of publication bias across studies.

### 3.7. Time Series Analysis

**Figure 6:**
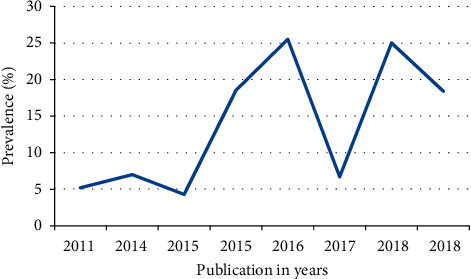
Time trend analysis of adult malaria prevalence in Ethiopia from 2011 to 2018.

The trend in malaria prevalence among included studies did not uniformly change within the ten years (2011–2018). From 2011 to 2014, the prevalence was characterized by a slight change in either direction (right and left). On the other hand, from 2016 to 2018, the prevalence showed an abrupt decrement and increment compared to the previous years (Figure 6).

## 4. Discussion

Despite the declining of malaria in Ethiopia, the disease is still a major public health concern, and it is one of the leading causes of morbidity and mortality among adult populations [34, 35], especially the productive age groups (15–45 years) were more exposed to *Plasmodium* infections in some areas [36]. This might be associated with respondents in these age groups who are likely to have lower malaria immunity than those in higher age groups. Moreover, higher exposure to outdoor activities of adults before bed time exposes more to the infection [37].

The current systematic review and meta-analysis was conducted using eight full-text articles to determine the pooled prevalence of malaria among adults in Ethiopia. Malaria causes serious complications in human, such as severe anaemia, acute renal failure, hypoglycemia [10], loss of productivity, school absenteeism, and other complications [38]. Therefore, the accurate malaria prevalence information is vital for the proper diagnosis, treatment, prevention, and policy preparation [39].

This systematic review and meta-analysis study showed that the pooled prevalence of malaria among adults in Ethiopia was 13.61%. This is much greater than the 2015 malaria indicator survey among the general population of the country, which results in 1.2% and 0.5% malaria prevalence by RDT and microscopy tests, respectively [40]. This study is nearly similar to systematic review and meta-analysis conducted among pregnant women in Ethiopia with the pooled prevalence of malaria 12.72% [41]. On the other hand, the result of this study was much lower than the previous research studies reported in Ethiopia among all age groups [39, 42].

The comparative decrement of malaria prevalence in the current study might be related to life style change, geographical area, economic status, the type of malaria diagnosis methods used, and the time at which the study conducted [33]. In addition to this, the malaria elimination and control program, such as proper use of long-lasting insecticide-treated nets (LLIN), insecticide residual spraying (IRS), introduction of rapid diagnostic tests at community level, and adaptation of artemisinin-based combination therapies (ACTs), might have led to a reduction in the burden of malaria in Ethiopia [27].

Subgroup analysis based on types of malaria cases showed that the prevalence of malaria among symptomatic and asymptomatic adults were 15.45% and 11.98%, respectively. The difference in the prevalence of malaria between the two groups is associated with parasite load; there might be higher parasite load among symptomatic cases than asymptomatic cases [41]. Similarly, regional subgroup analysis showed that the highest malaria prevalence was recorded in SNNPR (16.17%) followed by Oromia Regional State (13.25%) and the least was documented in Amhara Regional State (12.41%). This heterogeneity could be observed due to differences in methodology, sample size and sampling technique, and study participant characteristics. In addition, these variations could be explained by the existing differences in the environmental condition, rainfall, climate condition, residents' lifestyles, existence of swampy and irrigation areas, and the extent to which prevention and control measures are applied [41]. Moreover, the most likely reason for this variation is that some of the studies were obtained from the high malaria-endemic areas of the country, while others were obtained from medium and low malaria-risk areas. Furthermore, studies were conducted in different malaria transmission seasons, which means some studies were conducted during the high transmission periods while others were conducted during the least transmission seasons [33] which could be a significant contributing factor for the high variations [39]. On the other hand, the variations could be due to the presence of different topographic platforms that control the multiplication rate and diversity of malaria vectors [43].

The trend of malaria prevalence among studies included in this review did not uniformly change within the ten years (2010–2019) (Figure 6). However, this discrepancy in prevalence estimates over time could be due to the fact that malaria infection in Ethiopia is highly variable and unstable and the occurrence of epidemics over several locations (agroecological regions) of the country [39].

The overall pooled prevalence of *P. falciparum* (6.48%) and *P. vivax* (5.74%) parasites was found in proportions of 48.6% and 47.5%, respectively. The result of this study showed that *P. falciparum* and *P. vivax* have nearly equal contributions to the disease, malaria. This is similar to a study conducted in Batu town, Oromia Regional State [44]. On the other hand, the results contradicted to the previous reports in which *P. falciparum* proportion is greater than those of *P. vivax* in Ethiopia [23, 40, 42, 45]. In addition, it also contradicted the study that showed the prevalence of *P. vivax* is higher than *P. falciparum* [46–48]. Almost equal proportions of *P. falciparum* and *P. vivax* infections in our study indicate a shift in the trend of species composition in Ethiopia.

### 4.1. Limitations

Malaria is an important public health problem among adult individuals in Ethiopia. This study summarized the prevalence of malaria among adult in Ethiopia that can be an input for policymakers. Despite this, the present study has few limitations. First, a small number of articles included in this systematic review and meta-analysis could affect the pooled prevalence estimate. Second, almost all of the included studies were cross-sectional studies, and due to such reasons, the outcome variable could be affected by other confounding variables. Third, half of the included articles were obtained from the Oromia Regional State, whereas the other half was found to be in Amhara Regional State and SNNPR. This unequal distribution of articles throughout the country may affect the outcomes of this study. Fourth, no study obtained from Afar, Benishangul Gumuz, Gambella, Harari, and Tigray regions. These aforementioned limitations might affect the results reported in this review regarding the overall prevalence of malaria among adults in Ethiopia.

## 5. Conclusions

The current systematic review and meta-analysis showed that the pooled prevalence of malaria among adults was found to be greater than the general population and is nearly equal to pregnant women. Therefore, the existing prevention and control measures such as health education, usage of LLIN and application of IRS for controlling the vector, early diagnosis and treatment, and adaptation of artemisinin-based therapies should be adopted. In addition to this, Ethiopian public health institution and other responsible bodies should focus on the substantial reduction of mosquito-breading sites through community participation. Moreover, monitoring and mapping the distribution of insecticide resistance is appropriate in order to develop new vector control strategies.

## Data Availability

The data used and analyzed during the current study are available within the manuscript and supplementary materials.

## Supplementary Materials

Supplementary Materials Table S1: the nine criteria of the JBI quality assessment tool used to evaluate this prevalence review study. Table S2: the characteristics of 8 eligible studies included in this meta-analysis.
