# Supplementary material for: Prevalence of Malaria among Adults in Ethiopia: A Systematic Review and Meta-Analysis
Source: J Trop Med. 2021 Mar 4;2021:8863002. doi: 10.1155/2021/8863002 (PMC7952180; doi:10.1155/2021/8863002)
Supplement: Supplementary Materials — Table S1: the nine criteria of the JBI quality assessment tool used to evaluate this prevalence review study. Table S2: the characteristics of 8 eligible studies included in this meta-analysis. [file 8863002.f1.zip › 8863002.f1/Supplementary file 1.docx]

**Supplementary file 1**. Joanna Briggs Institute’s critical appraisal checklist for studies reporting prevalence data

| No | **Checklists** | **Yes** | **No** | **unclear** | **Not applicable** |
| --- | --- | --- | --- | --- | --- |
| 1 | Was the sample frame appropriate to address the target population? | 🖵 | 🖵 | 🖵 | 🖵 |
| 2 | Were study participants sampled in an appropriate way? | 🖵 | 🖵 | 🖵 | 🖵 |
| 3 | Was the sample size adequate? | 🖵 | 🖵 | 🖵 | 🖵 |
| 4 | Were the study subjects and the setting described in detail? | 🖵 | 🖵 | 🖵 | 🖵 |
| 5 | Was the data analysis conducted with sufficient coverage of the identified sample? | 🖵 | 🖵 | 🖵 | 🖵 |
| 6 | Were valid methods used for the identification of the condition? | 🖵 | 🖵 | 🖵 | 🖵 |
| 7 | Was the condition measured in a standard, reliable way for all participants? | 🖵 | 🖵 | 🖵 | 🖵 |
| 8 | Was there appropriate statistical analysis? | 🖵 | 🖵 | 🖵 | 🖵 |
| 9 | Was the response rate adequate, and if not, was the low response rate managed appropriately? | 🖵 | 🖵 | 🖵 | 🖵 |
