# Supplementary material for: Prevalence of Malaria among Adults in Ethiopia: A Systematic Review and Meta-Analysis
Source: J Trop Med. 2021 Mar 4;2021:8863002. doi: 10.1155/2021/8863002 (PMC7952180; doi:10.1155/2021/8863002)
Supplement: Supplementary Materials — Table S1: the nine criteria of the JBI quality assessment tool used to evaluate this prevalence review study. Table S2: the characteristics of 8 eligible studies included in this meta-analysis. [file 8863002.f1.zip › 8863002.f1/Supplementary file 2.docx]

**Supplementary file 2.** Characteristics of the 8 eligible studies included in this meta-analysis

| First author | Year | Region | Study group | Methods of diagnosis | Sample  n | Positive  n | *P. falciparum*  n (%) | *P. vivax*  n (%) | Mixed  n(%) | Total prevalence% (95% CI) |
| --- | --- | --- | --- | --- | --- | --- | --- | --- | --- | --- |
| Regasa *et al*., [[41](#_ENREF_41)] | 2014 | SNNPR | Suspected | Microscopy | 400 | 28 | 18 (4.5) | 7 (1.75) | 3 (0.75) | 7 (4.7-10.0) |
| Alemu *et al*.,[[42](#_ENREF_42)] | 2011 | Oromia | Suspected | Microscopy | 804 | 42 | 11(1.4) | 30(3.7) | 1(0.1) | 5.2(3.8-7.0) |
| Degefa *et al*., [[43](#_ENREF_43)] | 2015 | Oromia | Suspected | Microscopy | 604 | 112 | 58(9.6) | 52(8.6) | 2(0.3) | 18.5(15.5-21.9) |
| Delili *et al*.,[[25](#_ENREF_25)] | 2016 | SNNPR | Suspected | Microscopy | 422 | 106 | 27(6.4) | 76(18) | 3(0.7) | 25.8(21.1-29.5) |
| Fekadu *et al*.,[[28](#_ENREF_28)] | 2018 | Amhara | Suspected | RDT | 832 | 56 | 46(5.5) | 5(0.6) | 5(0.6) | 6.7(5.1-8.7) |
| Tadesse *et al*.,[[27](#_ENREF_27)] | 2018 | Oromia | Suspected | Microscopy | 810 | 204 | 92(11.4) | 110(13.6) | 2(0.2) | 25(22.2-28.3) |
| Aschale *et al*.,[[23](#_ENREF_23)] | 2018 | Amhara | Migrant | Microscopy | 385 | 71 | 50(13) | 7(1.8) | 14(3.6) | 18.4(14.7-22.7) |
| Alemayehu *et al*.,[[24](#_ENREF_24)] | 2015 | Oromia | Suspected | Microscopy | 3638 | 156 | 75(2.1) | 81(2.2) | 0(0) | 4.3(3.7-5.0) |
| Total |  |  |  |  | 7895 | 775 | 377 (6.48) | 368(5.74) | 30(0.47) | 13.6( 8.7- 18.5) |
